# Microbes, and What They Are Doing

**Published:** 1892-11

**Authors:** D. V. Beacock

**Affiliations:** Brockville, Ont.


ARTICLE III.

### MICROBES, AND WHAT THEY ARE DOING.

BY D. V. BEACOCK, BROCKVILLE, ONT.

There are three conditions requisite to produce septic fermentation, viz., warmth, moisture and microbes.

Pathogenic fermentation produces ptomaines. An open wound is a constant invitation to floating germs; these soon generate pus.

Now, what is pus? Ask any number of physicians or dentists, and note how the answers will vary. One says dead matter is pus. Another replies, pus is dead blood. One tells us it may be defined as the result of a pathological state, pyogenia. Another, the viscous matter of a sore, a well-known product of inflammation. Webster defines pus as the matter of an ulcer.

Dr. Thomas, in his medical dictionary, defines pus as a bland, cream-like fluid found in abscesses or on the surface of sores,

Dr. Robert Hunter, in his dictionary, states the word is from the Greek and Latin, and in the Sanscrit is called *puya*, meaning to stink. He says it consists of pus corpuscles, liquor puris, and other histological particles, and may be healthy or laudable, sanious, ichorous or watery, purulent or serous, and may become cheesy and ultimately calcify.

In Cleveland's dictionary we find pus defined as matter produced by suppuration, a cream-like fluid, heavier than water.

Virchow calls pus dead or destroyed tissue.

Dr. Black's is undoubtedly the best definition ever given us. He defines pus as the liquefaction of the plastic exudate, by the operation of microbes, death of the ameboid cells, from the changed chemical character of their matrix. Here we see the exudate or matrix of these ameboid cells so changed that it fails to support them; they die, and the resultant mass is pus. These cells are called white blood corpuscles or leucocytes when they are in the blood, when they are outside in the tissues, they are called ameboid or wandering cells. They pass into the tissues from the blood vessels by a process called diapedsis, which means a oozing through without rupturing the walls of the vessels confining them. These cells are the white blood corpuscles, or more properly undeveloped connective tissue walls, and one of their functions in nature is to repair injuries. The plastic exudate thrown out during the process of inflammation, forms the matrix in which these ameboid cells develop. They are always found imbedded in it, and it is absolutely essential to their final development into living tissue.

By ameboid properties is meant not only the capability of free movement, but the possession of a power which enables a cell to take foreign particles into its interior.

An exposed pulp is in a similar condition to an open wound, and both must be kept entirely free from contact with pus producing germs. If once they enter this plastic exudate, it begins to liquefy, its chemical character is changed, it fails to support the ameboid cells, consequently they die; they are then known as pus corpuscles.

In open wounds this process always takes place on the outer surface; these ameboid or undeveloped connective tissue cells continue to pile up in the form of living granulations, some of them floating away in the liquefying mass that ought to have formed their matrix. In this way the matrix or exudate is kept constantly filled with ameboid cells, tending to develop into healthy granulations.

On the contrary, the liquefaction of some of this plastic exudate carries off some of these cells in the form of pus.

If the former exceed the latter process, healing by what is commonly known as first intention takes place; if on the other hand, the latter process exceeds the former, destruction of tissue is the result.

The accumulation of the waste products of the pyogenic fungi occurs in the pus of abscesses, rendering it unfit for the continued growth of the fungi which produced it. The operation of the fungus thus becomes limited to the fresh exudates thrown in from the wound. This is in turn limited as the wall- of the wound become more solidly packed with ameboid cells, that is, living matter which is not so readily attacked. Microbes of pus formation cannot maintain themselves continuously in contact with living healthy tissue. This is a plain proof that vitality is one of the best germicides. All pus, no matter where found, whether upon the surface, in closed abscesses, or situated deep within the living tissues, is filled with micro-organisms.

The aim of the modern surgeon is to obtain the healing of wounds without suppuration. To this end he eliminates all micro-organisms, and uses dressings to prevent the access of germs to the wound. The intelligent dentist applies the same principle in his treatment of exposed pulps. Chronic and acute inflammations and abscesses of the mouth are due to the same pathological conditions which produce like results elsewhere in the body.

Modern aseptic dentistry consists in sterilization by germicides, dessication, etc. A fresh wound, if made aseptic, will heal by first intention; but if pyogenic germs are allowed to enter in any manner whatever, pus will be formed and trouble ensue, provided antiseptics are not carefully used.

In all those cases where the pulp chamber is opened for the first time, as in the removal of a living pulp, or a pulp destroyed by the operator, we should never have an abscess occur, indeed it should be impossible except through direct infection.

An intelligent physician or dentist can now do almost anything he pleases, providing he conforms to aseptic and antiseptic methods.

In this way bacteriology may be said to have revolutionized the theory and practice of dentistry and medicine.

Aseptic treatment means to preserve a clean wound from septic infection. Antiseptic treatment simply means the prevention of further extension of existing trouble. The one may be said to prevent fire, the other to extinguish it. For a similar reason antiseptics are not disinfectants; they do not destroy micro-organisms, they only prevent or inhibit their growth. A germicide may be all three, antiseptic, germicide and disinfectant.

To the physiologist, bacteria are subjects of the greatest interest. Only think of the occult manner in which they produce the deadly and poisonous ptomaines, the mysterious character of fermentation, which is in numerous instances produced by them, lactic fermentation or the souring of milk, ammoniacal fermentation, vinous fermentation, the rotting of fish, meat and other nitrogenous substances: in fact, all putrefaction is the result of the ceaseless activity of these countless organisms.

When we investigate or carefully examine bacteria and their doings, from a pathological standpoint, we reach the very climax of wonder, wars, pestilence and famine. In fact, nature's most dire cataclysm sinks into insignificance compared with the destructive work of these pathogenic and infinitesimal organisms. It is fortunate for the human race that only a small proportion of bacteria, comparatively speaking, are pathogenic; the great majority are benign, their great work being for good in the world's economy. In acting the part of scavengers, they simply return the elements of organization back to their original source with renewed activities for newer and higher combinations.

Every man among us lives by changes wrought in the chemical constituents of his environment. Each one of us is daily producing changes in quantities of chemical compounds known as food material, and constantly giving it back to the material world in chemical forms completely changed. The microbe is doing no more or no less.

The pathogenic germ is man's enemy, the benign germ is his friend. Bacteria are necessary as well as useful, for without them our farmers and gardeners would have little better than a desert or barren waste to till. Even our digestion is to a certain extent dependent on the family of benign germs, and millions occupy every portion of our bodies, no doubt for a beneficient purpose, although we may not realize it.

Paradoxical as the above may appear at first sight, it is nevertheless true that many of these germs are physiological. Pasteur isolated no less than seventeen different micro-organisms in the mouth; some of these dissolved albumen, caseine, and others converted starch into sugar. It therefore follows that the fermentative change they produce in food is a most important feature in digestion.

Even the very pus that these micro-organisms have been so persistent in elaborating has a beneficial purpose as a remedial process, such as granulation. etc., and frequently takes the place of far more morbid processes. It also affords a mechanical means of removing foreign bodies, *e. g.*, thorns, spinters, bits of broken glass, etc., from soft parts into which they may have been driven, and likewise in the formation of abscesses, may sometimes serve to eliminate morbid matter from the system.

All nature moves in a continuous change of cycles. Grass and herbs spring from the earth, air and water; herbiverous animals live and thrive on these, thus changing the constituents into other forms of food. These again are eaten by man and animals, and are again changed into other forms to be again transformed into other material, making food for microbes and finally returned to the earth from which they all originated. Thus we see the whole animal world may be said to be preying on each other; even one set of microbes are destroyed and eaten by others (phagocytism), and these again by others, so that Swift's couplet is quite applicable :—

> "The very fleas that do us tease,
> Have lesser fleas to bite them,
> And these again have lesser fleas
> And so *ad infinitum.*"

Out of the eight different processes by which the animal tissues are enabled to protect themselves against the action of bacteria, there are two which are very efficient, viz., phagocytism, and what may be denominated the bactericidal condition. Phagocytism, whether under normal or pathological conditions, is one of the manifestations of vis medicatrix nature. Under this condition, cellular activity prevents the development and increase of micro-organisms.

Under the latter a chemical condition is induced, which not only destroys microbes, reduces their nutrition, but retards their growth and multiplication.

It is by the activity of the ameboid cell or phagocyte that the gills and tails of tadpoles are removed during their metamorphosis.

Between the pyogenic microbe and the phagocyte there is a constant war even unto death.

Hess, to prove that the phagocyte cells were really aggressive in attacking pyogenic organisms, caused to be inserted, under the skin of a dog, a small capsule of glass with only a minute opening in one end. Into this capsule he had previously injected a quantity of Agar-Agar infected with staphlococci. The capsule, after a sufficient time, was removed from among the tissues. The phagocytes were found to be engorged with cocci.

You have all no doubt heard or read of Metchnicoff's vivid description of an abscess. He likens specific inflammation to a warfare, in which the invading army is represented by micro-organisms, and the resisting force by leucocytes. Even in details the analogy was maintained. Notice of the arrival of the invaders was telegraphed, so to speak, by the vaso-motor nerves; the line of communication, the avenues of mobilization, were represented by the blood vessels. The aim of the invader is to secure the territory, to multiply rapidly, to live at the expense of the host, and to manufacture and circulate substances injurious to him. The aim of the resisting forces is to encircle the enemy, inclose him, digest him, and render him inert in battle. Many phagocytes die in the process, and if in large numbers, the heaps of the slain represent pus. An abscess therefore is a battle ground. densely packed with dead bodies.

As dentists we have to admit that pyogenic fungi are ever present in the mouth, consequently every wound we inflict is in peril of becoming infected by them.

Dr. Miller says that every tooth extracted which is not performed under antiseptic precautions, is nothing less than an inoculation, and whether the subject proves refractory or not will depend upon a variety of circumstances, such as the size of the wound, resistance of the parts, the character and number of bacteria entering the wound, the health and vitality of the patient.

Dr. Sternberg says he found in his own mouth at all times, sufficient microbes in the saliva to kill a rabbit in twenty-eight hours after being injected.

Microbes produce disease by manufacturing substances by their physiological processes of growth and development, which is injurious to health. Many are found to be quite harmless, others are dangerous in the highest degree.

Microbes may be carried through the circulation to a focus of inflammation, and there set up a suppurating process. For instance, if the ear of an animal is injected with pus forming microbes, a wound in the extremities of the body may become infected through the circulation.

It is quite evident that microbes may easily become the cause of many of our diseases. For instance, a wandering corpuscle from some suppurating tissue, getting entangled in some debilitated part of the system, begins its work of generation, and thus boils, carbuncles, swellings, and many other serious troubles result. These cells or corpuscles are not the cause, it must be remembered, until they have become demoralized by microbes or ptomaines.

It is still a matter of doubt as to what and how these ptomaines or waste products are produced. In many cases they are the excreta of microbes themselves, in other cases they are the result of the splitting up of more complex substance, or coalescing of simpler bodies by the disturbance of molecular state of the compounds caused by the growth of the micro-organism. Waste products of microbes are analogous to the waste products of the other forms of life. In a large proportion of cases they are active poisons. They are *always* poisonous to the form of life that produced them, that is, providing they exceed certain proportions. Strange as it may appear from the above, it will be seen that microbes actually manufacture their own germicides, as certain substances which they elaborate are the excreta of germs which are poisonous to them, just as the excreta of any animal is poison to it.

Prof. Hamilton asks: What is the immediate cause of putrefaction, and of septicemia, or blood poisoning, if bacteria are not? and states his belief that the cause is the resultant products of bacteria, known as ptomaines, which have been found to be crystalline alkaloids.

Dr. Black mentions that he has often passed a platinum suture wire, after making it red-hot, to disinfect it, into a foul root canal, and then into stiff cultivating media, four or five inches, and has seen the development of microbes along the track of the wire from one end to the other. Now, he asks, if these organisms can be carried into stiff gelatine in this way with a perfectly smooth platinum wire, what may we expect from a barbed broach thrust through a foul root canal into the healthy tissue beyond?

Prof. Miller states, in looking over the literature of the subject, he had found fifty cases of death, resulting from abscesses caused by diseased teeth, or from dental operations performed without proper antiseptic precautions; and says, doubtless there have been hundreds of such cases, but the practitioner is not willing to have them made public. Serious results may also follow the wounding of the soft parts of the mouth, by the accidental slipping of germ laden burs, drills, excavators, etc., while working on the teeth. A young lady graduate had the misfortune to accidentally wound her finger while using the dental engine. The wound proved fatal.

You will, no doubt, be inclined to ask, What has all this to do with dentistry? I can only answer, very little, to those who think our profession consists in simply knowing how to manipulate the gas-bag, forceps and vulcanizer. On the other hand, it means a great deal to those who look upon dentistry as being a branch of the healing art. The mouth being the portal, or entrance to the system, it exerts a much greater influence over our general health than either patients or physicians are willing to admit. Only think for a moment, that the mouth may at any time become the focus of infection in many ways, and thus lay the foundation for some of the most dangerous diseases, and some of the worst of these may be brought about by the dentist's inability or carelessness. It behooves us as dentists to make ourselves well acquainted with the science of bacteriology, for the literature of medicine is *filled* with triumphal records of aseptic and antiseptic surgery.

Anyone who has occupied himself with this subject knows that the loss of appetite, nausea, and general ill-health are often brought about by improper attention to the mouth, causing a chronic state of putrefaction, the products being absorbed by the mucous membrane, with serious results to the general health, and these patients finally restored to good health by simply putting the mouth in a normal condition.

Tuberculosis is an infectious disease that is readily conveyed from one person to another, and is caused by a micro-organism which attacks the lungs. Expectoration follows; the bacilli are in the sputta, these may lodge in a decayed tooth, on the gums, or in other parts of the oral cavity. Has it ever occurred to any of you that these micro-organisms may be conveyed from the mouth of a consumptive to the mouth of a healthy person, for it is said that when direct inoculation occurs by means of an instrument in the hands of a dentist or physician, it is almost certain to prove fatal?

I sometimes think we do not realize the fearful responsibility resting upon us as dentists in regard to this matter. Let me illustrate: We use the gum lancet, or extract a tooth in a mouth in which there are specific ulcers; the instruments are covered with infecting pus; we wipe or clean them off, or at least we think we do, and imagine that they are clean, while in reality they are in the best possible condition for inoculation; an innocent lady takes her seat in your beautifully upholstered chair, her gums are lanced, or a tooth extracted with this same instrument. What is likely to be the result, I ask, of such a slip-shod performance? I will leave you to draw your own conclusions, and say nothing of the long train of *unfortunate consequences* that may follow, even to *succeeding generations.*

It cannot be too strongly impressed upon our minds that all instruments should be not merely cleansed, but thoroughly *sterilized* after use, or the next confiding patient may become inoculated.

We should ever remember that the law of asepsis rules every part of the great territory of antiseptic work, and in no department more than in dentistry.

If cleanliness is next to godliness anywhere, it is certainly doubly so in the mouth.—*Dominion Dent. Journal.*

